# Longitudinal Changes in Medial Meniscal Extrusion After ACL Injury and Reconstruction and Its Relationship With Cartilage Degeneration Assessed Using MRI-Based T1ρ and T2 Analysis

**DOI:** 10.1177/03635465241305734

**Published:** 2025-01-02

**Authors:** Shotaro Watanabe, Gabby B. Joseph, Dai Sato, Drew A. Lansdown, Julio Brandao Guimaraes, Thomas M. Link, Chunbong Benjamin Ma

**Affiliations:** † Department of Orthopaedic Surgery, University of California, San Francisco, San Francisco, California, USA; ‡ Department of Radiology and Biomedical Imaging, University of California, San Francisco, San Francisco, California, USA; § Department of Orthopaedic Surgery, Graduate School of Medical and Pharmaceutical Sciences, Chiba University, Center for Preventive Medical Sciences, Chiba University, Chiba, Japan; ‖ Department of Orthopaedic Surgery, Hokkaido University Graduate School of Medicine, Sapporo, Japan; ¶ Department of Musculoskeletal Radiology, Fleury Medicina e Saúde, São Paulo, Brazil; # Department of Radiology, Universidade Federal de São Paulo, São Paulo, Brazil; Investigation performed at the Department of Orthopaedic Surgery, University of California, San Francisco, California, USA

**Keywords:** anterior cruciate ligament injury, anterior cruciate ligament reconstruction, medial meniscal extrusion, posttraumatic osteoarthritis, quantitative magnetic resonance imaging

## Abstract

**Background:**

Anterior cruciate ligament (ACL) injury often leads to posttraumatic osteoarthritis (PTOA), despite ACL reconstruction (ACLR). Medial meniscal extrusion (MME) is implicated in PTOA progression but remains understudied after ACL injury and ACLR.

**Hypothesis/Purpose:**

It was hypothesized that MME would increase longitudinally after ACL injury and ACLR, with greater changes in the ipsilateral knee compared with the contralateral knee, leading to cartilage degeneration. The study aimed to assess MME 3 years after ACLR and its relationship with magnetic resonance imaging (MRI) T1ρ and T2 as cartilage degeneration markers.

**Study Design:**

Cohort study; Level of evidence, 2.

**Methods:**

MME and relative percentage of extrusion (RPE) were measured on 3 coronal slices of 3-dimensional fast spin-echo images and the mean values were used. T1ρ and T2 sequences were obtained and cartilage compositional measurements were performed using in-house developed software with MATLAB. Mixed models were used to assess the longitudinal changes and linear regression was used to assess the relationships between RPE and T1ρ and T2 values.

**Results:**

A total of 54 participants with unilateral ACL injuries underwent preoperative bilateral knee MRI. A total of 36 participants completed MR scans at 6 months and 3 years after ACLR. MME and RPE measurements demonstrated high reliability (ICC > 0.88 and > 0.91, respectively). The predicted values of MME and RPE from the mixed models showed that the ipsilateral side had significantly greater MME and RPE than the contralateral side at all 3 time points (*P* = .023 for MME; *P* = .013 for RPE at baseline; and *P* < .001 at 6 months and *P* < .001 at 3 years for both MME and RPE). The rate of change of MME and RPE on the ipsilateral side was significantly greater than that on the contralateral side (*P* < .001). Postoperative RPE was associated with T1ρ and T2 values in the posterior medial femoral condyle.

**Conclusion:**

MME and RPE obtained pre- and postoperatively after ACLR on the ipsilateral side were significantly greater than those on the contralateral side, and the longitudinal increases on the ipsilateral side were greater than those on the contralateral side. Postoperative RPE was significantly associated with cartilage degeneration in the posterior medial femoral condyle.

Anterior cruciate ligament (ACL) injury has been associated with posttraumatic osteoarthritis (PTOA). While ACL reconstruction (ACLR) is a common treatment for knee instability after ACL injury, PTOA may still develop in the long-term postoperative period.^29^ The risk factors for developing osteoarthritis (OA) in patients with ACL injury and ACLR are not yet fully understood. Medial meniscectomy is considered by some studies to be a significant risk factor for OA, particularly in the long-term follow-up.^3,7,17,29,32^ The meniscus plays a crucial role in distributing the load to the cartilage.^23^ It is important to note that loss of the load-distributing function of the meniscus can lead to OA progression, even without a meniscectomy. It has been reported that medial meniscal (MM) extrusion (MME) is an independent predictor of the development of tibiofemoral cartilage and subchondral bone lesions, as well as OA progression.^1,23,37,38,45^ MM posterior root tear is well known as a common cause of MME.^4,14^ MM root tears and extrusion were reported to play an important role in OA progression.^8,9^ However, there are many cases with OA progression from increased MME even in the absence of MM posterior root tears, and the natural history of knee joint degeneration concerning MME is complex and not completely clarified. MME increases with meniscal tears, degeneration, elongation of the meniscotibial ligament (MTL), and osteophyte formation, which may indicate the early stage of OA.^13,23^

There are only a few reports on MME with ACL injury and ACLR. One study reported that longitudinal meniscal injuries are significantly affected MME and not improved by repair.^16^ In ACLR without meniscal injury, MME has been reported to have a significant increase postoperatively.^14,33^ Another study reported no difference in MME preoperatively compared with a control group of volunteers with healthy knees.^33^ However, on the other hand, ACL-injured patients with concomitant MTL tears have been reported to have a higher incidence of MME.^30^ Based on these findings to date, it is not clear whether MME is more prevalent in the ACL-injured knee compared with the contralateral knee, and longitudinal changes using more than 2 time points are also unknown. Therefore, it is not certain when MME occurs in patients who had ACLR and how long it continues to progress. The relationship between MME and magnetic resonance imaging (MRI) T1ρ and T2 values—a quantitative assessment of cartilage degeneration that has been established as an early factor in PTOA—has not been investigated but is needed.

We hypothesized that ACL-injured knees would already have greater MME than the contralateral knee, that MME would progress faster in the ipsilateral compared with the contralateral knee over time after ACLR, and that MME would be associated with T1ρ and T2 values of the cartilage. This study aimed to observe MME longitudinally from the time of ACL injury to 3 years after ACLR, to compare it with the contralateral side, and to assess the relationship between MME and cartilage degeneration.

## Methods

This prospective study was approved by our institutional review board. All participants were included by providing their consent forms for the study.

### Patients

This cohort was recruited for the longitudinal evaluation of ACLR.^10,12,40-42,48^ Patients with traumatic unilateral ACL injuries between July 2011 and September 2014 who had preoperative bilateral knee MRI imaging were included. Patients who were unable or unwilling to consent to the study, had a history of previous knee trauma, had previous knee surgery, required meniscus or cartilage repair, had a joint inflammatory disease, or had OA were excluded. The sample size for this study was calculated based on our preliminary T1ρ data. We expect to observe significantly higher T1ρ values in the medial compartments of injured knees compared to control knees, particularly in the most weight-bearing compartment (3 from the medial femoral condyles (MFC) and 1 from the medial tibia (MT)). The sample size required for each subcompartment to reach a power of 80% at a significance level of .05 was 12, 9, 34, and 11—the calculations of T1ρ quantification were adjusted for 4 multiple comparisons using a Bonferroni correction. We therefore proposed to study 50 adult patients with ACL injuries at baseline, allowing for up to 20% drop-out rate during the follow-up to ensure enough power for the study. The contralateral knees were supposed to be used as controls. Participants with missing preoperative, 6-month, and 3-year bilateral knee MRIs were also excluded.

### Surgical Procedure

All ACLRs were performed by 1 of the 3 board-certified, fellowship-trained orthopaedic surgeons (C.B.M. and two other surgeons) at a single institution and were performed with an anatomic single-bundle ACLR with either hamstring tendon autografts or soft tissue allografts. Bone tunnels were drilled independently, with the femoral tunnel placed either through an anteromedial portal technique or an outside-in technique. All patients underwent the standardized postoperative rehabilitation protocol as previously described.^40^

### Data Source

Patient characteristics—such as age, sex, height, weight, body mass index (BMI), and preoperative waiting period—were extracted from medical records, and surgical information was extracted from surgical records. The MRI data were stored in a secure system at our institution, from which the data were used for analysis.

### MRI Acquisition

MRI was performed as previously described.^10,12,42^ All images were performed using a 3.0 T MRI scanner (General Electric) with an 8-channel knee coil (Invivo Inc). The imaging protocol included sagittal high-resolution 3-dimensional (3D) fast spin-echo (FSE) images (repetition time (TR)/echo time (TE), 1,500/25 msec; echo train length, 32; matrix, 384 × 384; field of view, 16 cm; slice thickness, 1 mm [interpolated into 0.5 mm]) to evaluate cartilage and meniscal morphology, and a sagittal T1ρ/T2 quantification sequence developed previously in our laboratory^28^ for assessing cartilage composition. The imaging parameters were as follows for T1ρ mapping: TR/TE, 8/3 msec; time of recovery, 1.2 sec; number of slices, 26; time of spin lock, 0/10/40/80 msec; spin-lock frequency, 500 Hz; field of view, 14 cm; matrix, 256 × 128; slice thickness, 4 mm; and views per segment, 64. The imaging parameters were as follows for T2 mapping: preparation TE = 0/13.7/27.3/54.7 msec; total acquisition time, 9 min 37 sec.

### MME Measurement

**Figure 1.**
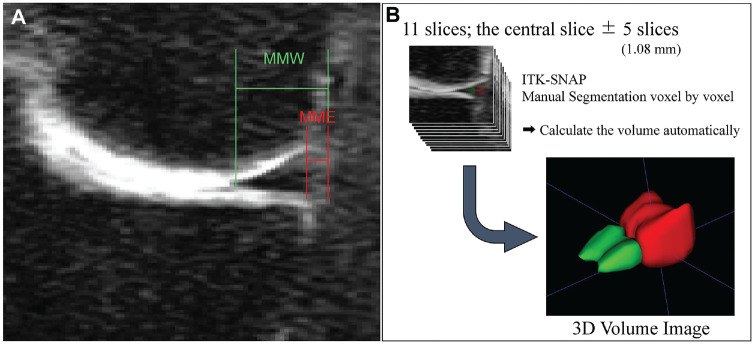
Measurement methods of MME and MM width. The coronal slice with the largest intercondylar ridge was defined as the central slice. (A) MME was defined as the length from the medial edge of the MM to the medial border of the tibia, excluding the osteophyte. The MMW was defined as the length of the entire MM from the medial edge of the MM to the central edge of the MM. (B) The 11 slices were annotated voxel by voxel for tibial MM (green) and MME (red). MM, medial meniscus; MME, medial meniscal extrusion; MMW, medial meniscal width; 3D, 3-dimensional.

MME measurements were performed on coronal reconstructions of the 3D-FSE images of both knees. First, we investigated the reproducibility and validity of the measurement method. The coronal slice with the largest intercondylar ridge was defined as the central slice, like that used in previous literature.^11,15,26^ We further defined the central slice as the slice containing the lowest point of the femoral condyle, because the inter-slice distance in our images was 0.27 mm, smaller than that in most of the previous literature, which was often 3 mm. The MME and MM width were measured on 11 slices—including 5 slices anterior and 5 slices posterior to the central slice. MME was defined as the length (mm) from the medial edge of the MM to the medial border of the tibia, excluding osteophytes. The MM width was defined as the length (mm) of the entire MM from the medial edge of the MM to the central edge of the MM (Figure 1A). The relative percentage of extrusion (RPE) was defined as MME divided by MM width and multiplied by 100 as previously reported.^19^ For further volume evaluation, the MM was segmented on those 11 slices, divided into MME and MM on the tibia, and the volume was measured on a voxel-by-voxel basis (Figure 1B). Each measurement was made with a software application named ITK-SNAP^47^ in 10 knees and performed by 2 orthopaedic surgeons with >10 years of experience (S.W. and D.S.) who were not the operating surgeons of this study cohort. Symmetrical combinations were examined in the central slice only, 5 combinations of 3 slices, 1 combination of 5 slices, and all 11 slices. We investigated the reproducibility and the validity in the several combinations of slices. From these results, we chose 1 measurement method for measuring all MRI scans in this study, considering time efficiency. MME and MM width lengths were measured by 1 orthopaedic surgeon (S.W.) who was not one of the operating surgeons of this study cohort on all MRI scans of both knees at baseline (preoperatively), 6 months postoperatively, and 3 years postoperatively to calculate the RPE.

### MRI Findings of Abnormalities in Knee Structures

All MRI examinations were evaluated in consensus by 1 radiologist with 15 years of experience (J.B.G.). The abnormalities included in the assessment were as follows: (1) ramp lesions, (2) lateral meniscus (LM) posterior root and other meniscus tears, (3) medial collateral ligament (MCL) injury, (4) posterolateral corner injury, and (5) MTL injury. An intrasubstantially situated complete thin, linear lesion between the posterior horn of the MM and the posteromedial capsule with an abnormally high fluid signal on fluid-sensitive sequences was defined as a ramp lesion on MRI (posterior medial meniscocapsular separation). Incomplete fluid interposition between the posterior horn of the MM and the capsule was also assessed. Ramp lesions were classified into 5 types using the classification of Thaunat et al^43^—type 1: meniscocapsular lesions; type 2: partial superior lesions; type 3: partial inferior or hidden lesions; type 4: complete tear in the red-red zone; and type 5: double tear. Meniscus root tears were defined as complete radial tears within 9 mm from the bony root attachment. MCL injuries were graded into 4 categories as follows: 0 = none; 1 = low grade; 2 = moderate grade; and 3 = high grade and rupture. Posterolateral corner injuries were graded into 4 categories as follows: 0 = none; 1 = low grade; 2 = moderate grade; and 3 = high grade and rupture. MTL injury was defined as abnormalities on MRI when there was a consensus that the ligament was poorly defined, attenuated, indistinct, or absent.^21,22^

### Cartilage T1ρ and T2 Relaxation Times Quantification

**Figure 2.**
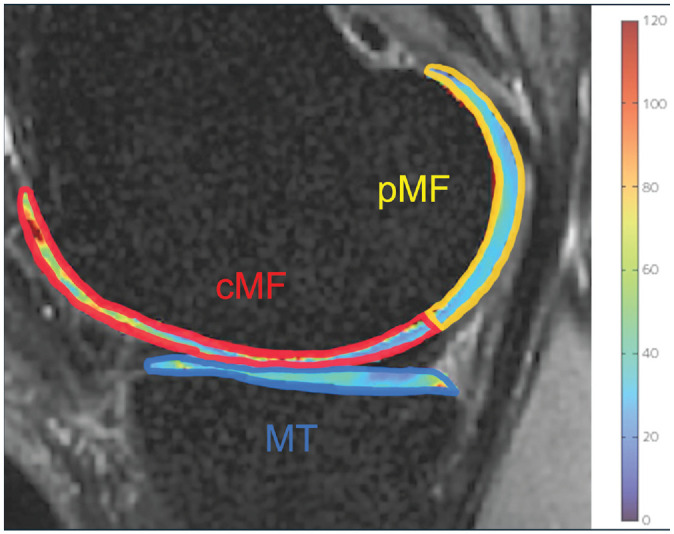
Segmentations of the MFC and tibial cartilage. MFC cartilage was subdivided into cMF (red area), pMF (yellow area), and MT (blue area) based on the meniscus. cMF, central medial femoral condyle; MFC, medial femoral condyle; MT, medial tibia; pMF, posterior medial femoral condyle.

The high-resolution 3D FSE images were downsampled in the sagittal direction and registered to the first echo of the T1ρ/T2 sequence. Postprocessing was done using in-house developed software with Matlab (Mathworks) integrated with the Elastix library for image registration.^20,39^ Cartilage was segmented semi-automatically on 3D FSE images using an algorithm based on edge detection and Bezier splines,^5^ MF, and MT. The MF was additionally subdivided into central medial femoral (cMF) and posterior medial femoral (pMF) subcompartments to examine the effects on each compartment of the knee (Figure 2). Registration was accomplished using an intensity-based multiresolution pyramidal approach and transferred from 3D FSE to the T1ρ and T2 maps pixel by pixel as previously described.^36^ The mean T1ρ and T2 values were calculated for each cartilage compartment.

### Statistical Analysis

Statistical analysis was performed using STATA Version 18 software (StataCorp LP). *P* < .05 was considered statistically significant.

We investigated the reproducibility in several combinations of slices by intraclass correlation coefficient (ICC) (1, 1) and ICC (2, 1), and the validity using the Spearman rank test with the RPE of MM volume.

Mixed models were used to assess the relationship between the joint side (predictor variable) and outcomes (MME and RPE of MM) over 36 months. These models included an interaction between the joint side and time point (baseline, 6 months, and 36 months) to explore how changes differ between sides over time. The models accounted for repeated measurements for each knee and person while adjusting for BMI, age, and sex.

Before performing the primary analyses, we tested the models for nonlinearity and confirmed that the changes in measured variables over time were linear. In the final models, the beta coefficients represent the difference in how outcomes (MME and RPE of MM) change over time between the ipsilateral and contralateral sides (difference in slopes). In addition, these mixed models were used to compare outcomes between the ipsilateral and contralateral sides at each time point—baseline, 6 months, and 36 months.

As explorational analyses, we investigated the meniscus and knee structures and compared the RPE with or without each injury using the Welch *t* test.

The relationships between the RPE (independent variable) and T1ρ and T2 values (dependent variables) at 6 months and 3 years postoperatively were assessed using linear regression adjusted for BMI and age. The T1ρ and T2 values of cartilage variables were the values of cMF, pMF, and MT (Figure 2). The medial 3 compartments were chosen because the load occurs on cMF and MT during a standing position and on pMF and MT during knee flexion.

## Results

### Patients Characteristics

A total of 54 patients were enrolled in this project, and 36 patients (16 women; 44%) completed the MRI scans at all 3 time points: baseline, 6 months postoperatively, and 3 years postoperatively. The mean age of the 36 patients at study enrollment was 31 ± 7.6 years, and their mean BMI was 23.9 ± 2.5. A total of 22 patients (61%) received a hamstring tendon autograft, whereas the remaining patients received a soft tissue allograft. The mean time between initial injury and preoperative MRI was 66.6 ± 49.2 days. The mean time between initial injury and surgery was 82.3 ± 56 days (Table 1).

**Table 1:** Baseline Characteristics^*a*^

Characteristics		
Sex	Male	20 (56)
	Female	16 (44)
Age, y		31 ± 7.6
BMI		23.9 ± 2.5
Side	Right	18 (50)
	Left	18 (50)
Time from injury to MRI, days		66.6 ± 49.2
Time from injury to surgery, days		82.3 ± 56
Graft	Hamstring tendon autograft	22 (61)
	Posterior tibialis allograft	13 (36)
	Hamstring tendon allograft	1 (3)

a Data are presented as mean ± SD or n (%). BMI, body mass index; MRI, magnetic resonance imaging.

### Measurement Method

Although the measurement using the 11 slices had the highest reproducibility, the measurement using the means of 3 slices also had sufficiently good reproducibility (Table 2). We decided to use the mean of 3 slices with central slice ± 4 slices (central slice ± 1.08 mm), which has a high ICC and a strong correlation with the volume measurement, considering time efficiency.

**Table 2:** The Reproducibility and Validity of the Measurement Method^*a*^

Measurement		ICC (1,1)	ICC (2,1)	Correlation With the Volume of Data
Volume data	MME	0.944	0.944	*r* = 1
	RPE	0.911	0.911	*P* < .000
Mean of 11 slices	MME	0.900	0.900	*r* = 0.990
	RPE	0.924	0.924	*P* < .001
Mean of 5 slices, central ± 2, 4 slices	MME	0.846	0.845	*r* = 0.982
	RPE	0.903	0.903	*P* < .001
Mean of 3 slices, central ± 5 slices	MME	0.859	0.858	*r* = 0.956
	RPE	0.907	0.906	*P* < .001
Mean of 3 slices,^*b*^ central ± 4 slices	MME	0.885	0.884	*r* = 0.971
	RPE	0.917	0.916	*P* < .001
Mean of 3 slices, central ± 3 slices	MME	0.691	0.689	*r* = 0.977
	RPE	0.858	0.857	*P* < .001
Mean of 3 slices, central ± 2 slices	MME	0.720	0.719	*r* = 0.953
	RPE	0.847	0.846	*P* < .001
Mean of 3 slices, central ± 1 slice	MME	0.848	0.853	*r* = 0.965
	RPE	0.920	0.920	*P* < .001
Central slice	MME	0.721	0.733	*r* = 0.919
	RPE	0.852	0.852	*P* < .001

a Spearman coefficients are denoted by *r*. Significant probabilities are denoted by *P*. Central ± 3 slices means using 3 slices—including the central slice, the slice located at 3 slices after the central slice, and the slice located at 3 slices before the central slice. Central, the central medial femoral condyle; ICC, intraclass correlation coefficients; MME, medial meniscal extrusion; RPE, relative percentage of extrusion.

b We used this method for the analysis in this study.

### Meniscal Extrusion and RPE

The results of the actual observed MME and RPE of MM for the ipsilateral and contralateral side at each time point are shown in Table 3. The predicted values of MME and RPE of MM from the mixed models are also presented in Table 3. At all 3 time points, the ipsilateral side had significantly greater MME and the RPE of MM than the contralateral side (*P* = .023 for MME; *P* = .013 for RPE of MM at baseline; and *P* < .001 at 6 months postoperatively and *P* < .001 at 3 years postoperatively for both MME and RPE) (Table 3).

**Table 3:** MME and RPE^*a*^

	Ipsilateral Actual Value, Mean ± SD	Contralateral Actual Value, Mean ± SD	Ipsilateral Predicted Value (95% CI)	Contralateral Predicted Value (95% CI)	*P*
MME, mm					
Baseline	2.10 ± 0.37	1.99 ± 0.36	2.13 (2.01-2.24)	1.98 (1.86-2.10)	**.023**
6 months	2.25 ± 0.45	2 ± 0.29	2.21 (2.10-2.32)	1.99 (1.88-2.10)	**<.001**
3 years	2.62 ± 0.51	2.03 ± 0.46	2.63 (2.49-2.76)	2.03 (1.89-2.16)	**<.001**
RPE of MM, %					
Baseline	32.1 ± 6.4	30.1 ± 6.6	31.8 (30.1-33.6)	29.6 (27.8-31.4)	**.013**
6 months	32.8 ± 7.2	29.3 ± 5.5	33.0 (31.3-34.6)	29.8 (28.1-31.5)	**<.001**
3 years	38.5 ± 9.3	31 ± 7.9	38.5 (36.6-40.5)	30.9 (28.9-32.8)	**<.001**

a Each value is a predicted value from the mixed model and may differ from an actual observed value. The *P* values are calculated using a mixed model. Bold *P* values represent a significant difference (*P* < .05). Mixed models are adjusted for BMI, sex, and age.

BMI, body mass index; MM, medial meniscal; MME, medial meniscal extrusion; RPE, relative percentage of extrusion.

**Figure 3.**
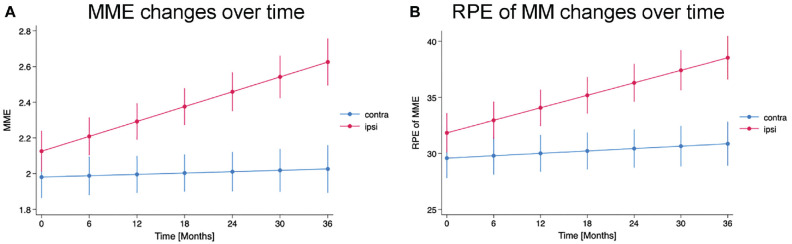
Changes in predicted MME and RPE of MM for each side over time. Time (from 0 to 36, in 6-unit increments) is plotted on the x-axis, while the predicted values of (A) MME and (B) RPE of MM are displayed on the y-axis. The predicted values are calculated based on a mixed-effects model, with 95% CIs shown on the graph. Each data point is a predicted value from the model and may differ from actual observed values. BMI, sex, and age are incorporated into the model as statistical adjustment factors. BMI, Body mass index; contra, contralateral; ipsi, ipsilateral; MM, medial meniscal; MME, medial meniscal extrusion; RPE, relative percentage of extrusion.

The mixed model shows that the rate of change of MME on the ipsilateral side is significantly greater than that of the contralateral side (coefficient, 0.013 [the difference in slope between the sides] [95% CI, 0.007-0.018]; *P* < .001,) (Figure 3A). The mixed linear model also shows the rate of change of the RPE of MM on the ipsilateral side is significantly greater than that of the contralateral side (coefficient, 0.151 [95% CI, 0.082-0.219]; *P* < .001) (Figure 3B).

The mixed model shows that the rate of change of MME on the ipsilateral side is significantly greater than that of the contralateral side (coefficient, 0.013 [the difference in slope between the sides] [95% CI, 0.007-0.018]; *P* < .001) (Figure 3A). The mixed linear model also shows the rate of change of the RPE of MM on the ipsilateral side is significantly greater than that of the contralateral side (coefficient, 0.151 [95% CI, 0.082-0.219]; *P* < .001) (Figure 3B).

### MRI Findings of Abnormalities in Knee Structures

Ramp lesions with type ≥3 (regarded as unstable) were found in 12 knees (33.3%), LM posterior root tears in 7 (19.4%) knees, MM tears in 22 (61.1%) knees, and LM tears in 15 knees (41.7%). Posterolateral corner injuries with grades of ≥2 were observed in 14 (38.9%) knees, MCL injuries with grade 3 in 4 (11.1%) knees, and MTL injuries in 8 (22.2%) knees. The preoperative RPE of MM showed no significant differences between knees with and without injury (Table 4).

**Table 4:** RPE in Knees with and without Injuries on MRI Scans^*a*^

			RPE, Mean (SD)		
Injuries		n (%)	With	Without	*P*
Ramp lesion	None	20 (55.5)			
	Type 1	0			
	Type 2	4 (11.1)			
	Type 3	0			
	Type 4	12 (33.3)			
	Type 5	0			
Ramp lesion type ≥3		12 (33.3)	32.8 (2.3)	31.8 (1.2)	.70
MM tear		22 (61.1)	32.2 (1.5)	32.0 (1.4)	.94
LM posterior root tear		7 (19.4)	30.1 (2.1)	32.6 (1.2)	.33
LM tear		15 (41.7)	32.6 (1.6)	31.8 (1.5)	.73
MM or LM tear		29 (80.6)	32.8 (1.3)	29.3 (1.5)	.09
PLC injury	Grade 0	15 (41.7)			
	Grade 1	7 (19.4)			
	Grade 2	11 (30.6)			
	Grade 3	3 (8.3)			
PLC injury with grade ≥2		14 (38.9)	33.6 (1.7)	31.2 (1.4)	.27
MCL injury with grade 3		4 (11.1)	34.8 (4.2)	31.8 (1.1)	.53
MTL injury		8 (22.2)	35.6 (2.8)	31.1 (1.1)	.16

a Ramp lesions were classified into 5 types—type 1: meniscocapsular lesions; type 2: partial superior lesions; type 3: partial inferior or hidden lesions; type 4: complete tear in the red-red zone; and type 5: double tear. Posterolateral corner injuries were divided into 4 grades—0 = none; 1 = low grade; 2 = moderate grade; and 3 = high grade and rupture). MCL injuries were divided into 4 grades—0 = none; 1 = low grade; 2 = moderate grade; and 3 = high grade and rupture.

LM, lateral meniscus; MCL, medial collateral ligament; MM, medial meniscus; MTL, meniscotibial ligament; PLC, posterolateral corner; RPE, relative percentage of extrusion.

### MME and Cartilage Degeneration Evaluated by MRI T1ρ and T2 Values

The RPE at 6 months postoperatively correlated positively with the T2 value in pMF at 6 months. The RPE at 3 years postoperatively also correlated positively with T1ρ and T2 values in pMF at 3 years (Table 5). No significant differences were observed in the other compartments.

**Table 5:** Relationships Between the RPE of MM and the T1ρ and T2 Value in Cartilage Areas after ACLR^*a*^

Outcome Variables	RPE of MM	Coefficient	SD	*t*-Value	*P*
6 months postoperatively					
T2 in pMF	Constant	32.3	5.66	5.7	<.001
(*R*^2^, 0.189)	RPE at 6 months	0.176	0.722	2.44	.021
3 years postoperatively					
T1ρ in pMF	Constant	47.54	5.09	9.34	<.001
(*R*^2^, 0.21)	RPE at 3 years	0.111	0.511	2.17	.038
T2 in pMF	Constant	35.8	4.06	8.88	<.001
(*R*^2^, 0.30)	RPE at 3 years	0.125	0.408	3.07	.004

a Regression analyses were performed with the T1ρ or T2 value of the area as outcome variables, and the RPE of MM, BMI, and age as independent variables. The RPE of MM had significant correlations only with pMF as shown above. pMF is defined in Figure 1 as pMF. No significant differences were observed in the other compartments. BMI, body mass index; MF, medial femoral; MME, medial meniscal extrusion; pMF, posterior medial femoral; RPE, relative percentage of extrusion.

## Discussion

The MME and RPE of MM preoperatively, 6 months postoperatively, and 3 years postoperatively were greater on the ACL-injured knee than on the contralateral side and increased longitudinally from preoperatively to 3 years postoperatively. In a mixed-effect model that adjusted for age, sex, and BMI, the slope of the increase in MME and RPE was significantly greater for the ACL-injured knee than for the contralateral knee. Postoperative RPE of MM was significantly associated with T1ρ and T2 values of the cartilage in the pMFC.

In terms of the measurement method used in this study, the mean of 3 slices was used to improve the reproducibility of the measurement, and the ICC of the RPE of MM was higher than that of MME. Furthermore, this method showed a strong correlation with MME volume, indicating that it is useful as a measurement method. Most reports use the coronal slice with the largest intercondylar ridge or the coronal slice with the largest MME.^11,15,26^ Jones et al^15^ reported that the former provides a more accurate MME. However, many reports of both methods use images taken at a 3 mm interslice distance, which is hardly an accurate definition of the slices used.^11,26^ Therefore, in the present study, we were able to improve reproducibility by using the lowest point of the medial condyle included in the slices with the largest intercondylar ridge. Some papers use MME measurements by volume.^18,34,46^ However, it is difficult to generalize considering the time efficiency. In this study, we also evaluated the validity of the correlation with volumetric evaluation using 11 slices of the cMFC and searched for the best measurement method. The use of RPE could contribute to minimizing measurement error. We obtained higher reproducibility with RPE than with absolute MME.

Only a few studies on MME in ACLR have been reported. Katagiri et al^16^ reported that longitudinal meniscal tears correlated with MME in ACLR and that meniscal extrusion persisted even after meniscal repair.^16^ The present study examined MME in ACLR, excluding meniscal repair. A few other studies have reported MME after ACLR, excluding meniscal repair. Narazaki et al^33^ reported greater MME in post-ACLR knees compared with normal knees in the control group. However, they reported no difference between normal knees and preoperative ACLR knees.^33^ Hada et al^14^ found MME (>3 mm) in 16.7% and 26.7% of patients before and after ACLR, respectively, and MME was greater postoperatively (7.6 months after ACLR) than preoperatively. Our study also showed similar results with the progression of MME, and this may continue for 3 years postoperatively. MME may be one of the most important risk factors for knee OA progression after ACLR without meniscal injury. The present study has another distinctive strong point in addition to the examination at 3 longitudinal time points up to 3 years later, which is the presence of the contralateral side MRI at each time point. Comparing the sides revealed that the MME of the ACL-injured knee was already greater than that of the healthy side at the time of pre-ACLR.

In our cases, we found no difference in RPE between knees with and without meniscal injuries, posterolateral corner injuries, MCL injuries with grade 3, and MTL injuries (Table 4). Krych et al^21^ reported that MTL injury was involved in knees with large MME but no meniscal injury or knee trauma. These authors also reported that in patients with medial knee pain, the meniscus was extruded with its roots intact, resulting in a progressive tear of the posterior root.^22^ The MTL is a critical stabilizing tissue and contributes to the stability of the meniscus.^35^ Biomechanical studies have also reported that MTL insufficiency resulted in increased loading on the MM posterior root.^31^ Mariani et al^30^ reported that ACL-injured patients with concomitant MTL tears had a higher incidence of large MME (>3 mm). In our study, the preoperative MME was already greater in the ACL-injured knee than the contralateral side; however, there were no significant differences in preoperative MME with or without other injuries in ACL injuries knees—including MTL injury. Multiple analyses with large numbers of cases may be needed to detect the effects of the injuries on MME progression. Furthermore, it has been reported that preoperative intra-articular inflammatory biomarkers are linked to cartilage degeneration after injury.^25^ Consequently, it may be also necessary to investigate the relationship between elevated MME and these biomarkers in a prospective study.

The T1ρ and T2 values of cartilage have been investigated in knees after ACLR and are useful for early detection of PTOA.^2,6,10,12,24,27,40-42,44,48^ However, the cause of the prolonged T1ρ and T2 relaxation time of the cartilage is not completely known. Although MM resection is a known risk factor for OA after ACLR and cartilage damage after MM posterior root tear is also well known, it is important to note that there are no reports on the association between MME and cartilage degeneration using T1ρ and T2 values. In the present study, the association between RPE and T1ρ and T2 was investigated, and it was found that RPE was associated with cartilage degeneration. This finding is a significant contribution not only to the prediction of post-ACLR outcomes but also to the elucidation of progression of osteoarthritis.

The study has some limitations that should be considered. First, the number of cases analyzed was limited. However, it should be emphasized that the study still provides valuable and useful information because it analyzed ACLRs without meniscal repairs at 3 time points using MRI 3D FSE images, and cartilage T1ρ and T2 values were available. Second, the MRI images were not acquired under load. Nevertheless, it is important to acknowledge that the results of this study using these images are still meaningful, as most MRI scans used in clinical practice are unloaded. If weightbearing MRI were to become more widely used in clinical practice in the future, it may be worth considering weightbearing imaging in the study design. Third, the procedures were performed by 3 surgeons and with either autologous hamstring tendons or allografts, which may have influenced the data. In addition, this study only included results from the soft tissue ACLR technique, and there was no comparison to other graft types. Fourth, MRI was performed between the ACL injury and the reconstruction surgery, often about a week before the surgery, thus the time between injury and preoperative MRI scan was variable, potentially affecting the progression of MME during this time. Finally, there is uncertainty regarding whether MME increases more than 3 years after surgery, and it is unclear from this study whether there is an association between cartilage degeneration and OA in the future. While this study showed an association between MME and cartilage degeneration for the first time, it is important to conduct long-term follow-ups to gain a better understanding of the situation.

By excluding cases of meniscal repairs, it may be possible to obtain a more generalized effect of ACL injury and ACLR that can be accepted as fact. This study provided a basis for all future conversations regarding MME after ACLR. Future research should explore the causes of increased MME in ACL-injured knees, how to repair MME during reconstructive surgery, and whether subsequent MME progression can be prevented if repair can be achieved.

## Conclusion

After ACLR, MME and RPE obtained preoperatively, 6 months, and 3 years postoperatively were significantly greater on the ipsilateral side than on the contralateral side, and the longitudinal increases on the ipsilateral side were greater than those on the contralateral side. The postoperative RPE of MM was significantly associated with T1ρ and T2 values of the cartilage in the pMFC. New insights have been gained into the progression of MME in patients undergoing ACLR and into the associations between cartilage T1ρ and T2 values and MME progression. These findings may be significant in understanding the development of OA after ACLR.

## References

[1] Adams JG McAlindon T Dimasi M Carey J Eustace S. Contribution of meniscal extrusion and cartilage loss to joint space narrowing in osteoarthritis. Clin Radiol. 1999;54(8):502-506. 10484216 10.1016/s0009-9260(99)90846-2

[2] Amano K Li AK Pedoia V , et al. Effects of surgical factors on cartilage can be detected using quantitative magnetic resonance imaging after anterior cruciate ligament reconstruction. Am J Sports Med. 2017;45(5):1075-1084. 28768432 10.1177/0363546516677794

[3] Barenius B Ponzer S Shalabi A Bujak R Norlén L Eriksson K. Increased risk of osteoarthritis after anterior cruciate ligament reconstruction: a 14-year follow-up study of a randomized controlled trial. Am J Sports Med. 2014;42(5):1049-1057. 24644301 10.1177/0363546514526139

[4] Bhatia S LaPrade CM Ellman MB LaPrade RF. Meniscal root tears: significance, diagnosis, and treatment. Am J Sports Med. 2014;42(12):3016-3030. 24623276 10.1177/0363546514524162

[5] Carballido-Gamio J Bauer JS Stahl R , et al. Inter-subject comparison of MRI knee cartilage thickness. Med Image Anal. 2008;12(2):120-135. 17923429 10.1016/j.media.2007.08.002 PMC2838773

[6] Casula V Tajik BE Kvist J , et al. Quantitative evaluation of the tibiofemoral joint cartilage by T2 mapping in patients with acute anterior cruciate ligament injury vs contralateral knees: results from the subacute phase using data from the NACOX study cohort. Osteoarthritis Cartilage. 2022;30(7):987-997. 35421548 10.1016/j.joca.2022.02.623

[7] Chalatsis G Mitrousias V Siouras A , et al. Long-term quality of life in patients after ACL reconstruction with concomitant meniscal injury treatment: patient-reported outcomes at minimum 10-year follow-up. Orthop J Sports Med. 2023;11(6):23259671231177280. 10.1177/23259671231177279 PMC10280537 37347018

[8] Foreman SC Liu Y Nevitt MC , et al. Meniscal root tears and extrusion are significantly associated with the development of accelerated knee osteoarthritis: data from the osteoarthritis initiative. Cartilage. 2021;13(suppl 1):S239-s248. 10.1177/1947603520934525 PMC8808926 32567341

[9] Foreman SC Neumann J Joseph GB , et al. Longitudinal MRI structural findings observed in accelerated knee osteoarthritis: data from the Osteoarthritis Initiative. Skeletal Radiol. 2019;48(12):1949-1959. 31209509 10.1007/s00256-019-03242-9 PMC6814533

[10] Friedman JM Su F Zhang AL , et al. Patient-reported activity levels correlate with early cartilage degeneration after anterior cruciate ligament reconstruction. Am J Sports Med. 2021;49(2):442-449. 33395319 10.1177/0363546520980431

[11] Gale DR Chaisson CE Totterman SM Schwartz RK Gale ME Felson D. Meniscal subluxation: association with osteoarthritis and joint space narrowing. Osteoarthritis Cartilage. 1999;7(6):526-532. 10558850 10.1053/joca.1999.0256

[12] Gong J Pedoia V Facchetti L Link TM Ma CB Li X. Bone marrow edema-like lesions (BMELs) are associated with higher T1ρ and T2 values of cartilage in anterior cruciate ligament (ACL)-reconstructed knees: a longitudinal study. Quant Imaging Med Surg. 2016;6(6):661-670. 28090444 10.21037/qims.2016.12.11 PMC5219965

[13] Hada S Ishijima M Kaneko H , et al. Association of medial meniscal extrusion with medial tibial osteophyte distance detected by T2 mapping MRI in patients with early-stage knee osteoarthritis. Arthritis Res Ther. 2017;19(1):201. 28899407 10.1186/s13075-017-1411-0 PMC5596458

[14] Hada S Kaneko H Liu L , et al. Medial meniscus extrusion is directly correlated with medial tibial osteophyte in patients received reconstruction surgery for anterior cruciate ligament injury: a longitudinal study. Osteoarthr Cartil Open. 2022;4(4):100320. 36474799 10.1016/j.ocarto.2022.100320 PMC9718326

[15] Jones LD Mellon SJ Kruger N Monk AP Price AJ Beard DJ. Medial meniscal extrusion: a validation study comparing different methods of assessment. Knee Surg Sports Traumatol Arthrosc. 2018;26(4):1152-1157. 28523339 10.1007/s00167-017-4544-4 PMC5876269

[16] Katagiri H Miyatake K Nakagawa Y , et al. The effect of a longitudinal tear of the medial meniscus on medial meniscal extrusion in anterior cruciate ligament injury patients. Knee. 2019;26(6):1292-1298. 31519329 10.1016/j.knee.2019.07.019

[17] Keays SL Newcombe PA Bullock-Saxton JE Bullock MI Keays AC. Factors involved in the development of osteoarthritis after anterior cruciate ligament surgery. Am J Sports Med. 2010;38(3):455-463. 20051501 10.1177/0363546509350914

[18] Kim C Bin SI Lee BS , et al. Volumetric assessment of extrusion in medial meniscus posterior root tears through semi-automatic segmentation on 3-tesla magnetic resonance images. Orthop Traumatol Surg Res. 2020;106(5):963-968. 32782171 10.1016/j.otsr.2020.02.020

[19] Kim JY Bin SI Kim JM Lee BS Oh SM Park MH. Tear gap and severity of osteoarthritis are associated with meniscal extrusion in degenerative medial meniscus posterior root tears. Orthop Traumatol Surg Res. 2019;105(7):1395-1399. 31575505 10.1016/j.otsr.2019.09.015

[20] Klein S Staring M Murphy K Viergever MA Pluim JPW . Elastix: a toolbox for intensity-based medical image registration. IEEE Trans Med Imaging. 2010;29(1):196-205. 19923044 10.1109/TMI.2009.2035616

[21] Krych AJ Bernard CD Leland DP , et al. Isolated meniscus extrusion associated with meniscotibial ligament abnormality. Knee Surg Sports Traumatol Arthrosc. 2020;28(11):3599-3605. 31332493 10.1007/s00167-019-05612-1

[22] Krych AJ LaPrade MD Hevesi M , et al. Investigating the chronology of meniscus root tears: do medial meniscus posterior root tears cause extrusion or the other way around? Orthop J Sports Med. 2020;8(11):2325967120961368. 10.1177/2325967120961368 PMC7645763 33209944

[23] Langhans MT Lamba A Saris DBF Smith P Krych AJ. Meniscal extrusion: diagnosis, etiology, and treatment options. Curr Rev Musculoskelet Med. 2023;16(7):316-327. 37191818 10.1007/s12178-023-09840-4 PMC10356705

[24] Lansdown DA Allen C Zaid M , et al. A comprehensive in vivo kinematic, quantitative MRI and functional evaluation following ACL reconstruction—a comparison between mini-two incision and anteromedial portal femoral tunnel drilling. Knee. 2015;22(6):547-553. 25982298 10.1016/j.knee.2014.12.005 PMC4472567

[25] Lattermann C Jacobs CA Proffitt Bunnell M , et al. A multicenter study of early anti-inflammatory treatment in patients with acute anterior cruciate ligament tear. Am J Sports Med. 2017;45(2):325-333. 28146402 10.1177/0363546516666818

[26] Lee DH Kim TH Lee SH Kim CW Kim JM Bin SI. Evaluation of meniscus allograft transplantation with serial magnetic resonance imaging during the first postoperative year: focus on graft extrusion. Arthroscopy. 2008;24(10):1115-1121. 19028163 10.1016/j.arthro.2008.01.016

[27] Li X Kuo D Theologis A , et al. Cartilage in anterior cruciate ligament-reconstructed knees: MR imaging T1{rho} and T2—initial experience with 1-year follow-up. Radiology. 2011;258(2):505-514. 21177392 10.1148/radiol.10101006 PMC3029884

[28] Li X Wyatt C Rivoire J , et al. Simultaneous acquisition of T1ρ and T2 quantification in knee cartilage: repeatability and diurnal variation. J Magn Reson Imaging. 2014;39(5):1287-1293. 23897756 10.1002/jmri.24253 PMC3844010

[29] Lohmander LS Englund PM Dahl LL Roos EM. The long-term consequence of anterior cruciate ligament and meniscus injuries: osteoarthritis. Am J Sports Med. 2007;35(10):1756-1769. 17761605 10.1177/0363546507307396

[30] Mariani PP Torre G Battaglia MJ. The post-traumatic meniscal extrusion, sign of meniscotibial ligament injury. A case series. Orthop Traumatol Surg Res. 2022;108(3):103226. 35123034 10.1016/j.otsr.2022.103226

[31] Melugin HP Brown JR Hollenbeck JFM , et al. Meniscotibial ligament insufficiency increases force on the posterior medial meniscus root. Am J Sports Med. 2023;51(13):3502-3508. 37681506 10.1177/03635465231194606

[32] MOON Knee Group, Spindler KP Huston LJ , et al. Ten-year outcomes and risk factors after anterior cruciate ligament reconstruction: a MOON longitudinal prospective cohort study. Am J Sports Med. 2018;46(4):815-825. 29543512 10.1177/0363546517749850 PMC6036619

[33] Narazaki S Furumatsu T Tanaka T , et al. Postoperative change in the length and extrusion of the medial meniscus after anterior cruciate ligament reconstruction. Int Orthop. 2015;39(12):2481-2487. 25693884 10.1007/s00264-015-2704-z

[34] Okazaki Y Furumatsu T Miyazawa S , et al. A novel suture technique to reduce the meniscus extrusion in the pullout repair for medial meniscus posterior root tears. Eur J Orthop Surg Traumatol. 2019;29(8):1805-1809. 31350648 10.1007/s00590-019-02513-4

[35] Paletta GA Jr Crane DM Konicek J , et al. Surgical treatment of meniscal extrusion: a biomechanical study on the role of the medial meniscotibial ligaments with early clinical validation. Orthop J Sports Med. 2020;8(7):2325967120936672. 32775474 10.1177/2325967120936672 PMC7391441

[36] Pedoia V Li X Su F Calixto N Majumdar S. Fully automatic analysis of the knee articular cartilage T1ρ relaxation time using voxel-based relaxometry. J Magn Reson Imaging. 2016;43(4):970-980. 26443990 10.1002/jmri.25065 PMC5018211

[37] Puig L Monllau JC Corrales M Pelfort X Melendo E Cáceres E. Factors affecting meniscal extrusion: correlation with MRI, clinical, and arthroscopic findings. Knee Surg Sports Traumatol Arthrosc. 2006;14(4):394-398. 16163556 10.1007/s00167-005-0688-8

[38] Raynauld JP Martel-Pelletier J Berthiaume MJ , et al. Long term evaluation of disease progression through the quantitative magnetic resonance imaging of symptomatic knee osteoarthritis patients: correlation with clinical symptoms and radiographic changes. Arthritis Res Ther. 2005;8(1):1-12. 10.1186/ar1875 PMC1526551 16507119

[39] Shamonin DP Bron EE Lelieveldt BP , et al. Fast parallel image registration on CPU and GPU for diagnostic classification of Alzheimer's disease. Front Neuroinform. 2014;7:50. 24474917 10.3389/fninf.2013.00050 PMC3893567

[40] Shimizu T Samaan MA Tanaka MS , et al. Abnormal biomechanics at 6 months are associated with cartilage degeneration at 3 years after anterior cruciate ligament reconstruction. Arthroscopy. 2019;35(2):511-520. 30473456 10.1016/j.arthro.2018.07.033 PMC6361700

[41] Su F Hilton JF Nardo L , et al. Cartilage morphology and T1ρ and T2 quantification in ACL-reconstructed knees: a 2-year follow-up. Osteoarthritis Cartilage. 2013;21(8):1058-1067. 23707754 10.1016/j.joca.2013.05.010 PMC3752987

[42] Su F Pedoia V Teng HL , et al. The association between MR T1ρ and T2 of cartilage and patient-reported outcomes after ACL injury and reconstruction. Osteoarthritis Cartilage. 2016;24(7):1180-1189. 26850823 10.1016/j.joca.2016.01.985 PMC4907855

[43] Thaunat M Fayard JM Guimaraes TM Jan N Murphy CG Sonnery-Cottet B. Classification and surgical repair of ramp lesions of the medial meniscus. Arthrosc Tech. 2016;5(4):e871-e875. 10.1016/j.eats.2016.04.009 PMC5040630 27709051

[44] Theologis AA Kuo D Cheng J , et al. Evaluation of bone bruises and associated cartilage in anterior cruciate ligament-injured and -reconstructed knees using quantitative t(1ρ) magnetic resonance imaging: 1-year cohort study. Arthroscopy. 2011;27(1):65-76. 21035995 10.1016/j.arthro.2010.06.026 PMC3011041

[45] Wang Y Wluka AE Pelletier JP , et al. Meniscal extrusion predicts increases in subchondral bone marrow lesions and bone cysts and expansion of subchondral bone in osteoarthritic knees. Rheumatology. 2010;49(5):997-1004. 20181669 10.1093/rheumatology/keq034

[46] Watanabe G Hoshi K Kurose Y Gamada K. High validity of measuring the width and volume of medial meniscal extrusion three-dimensionally using an MRI-derived tibial model. J Exp Orthop. 2020;7(1):1. 31900597 10.1186/s40634-019-0216-2 PMC6942059

[47] Yushkevich PA Piven J Hazlett HC , et al. User-guided 3D active contour segmentation of anatomical structures: significantly improved efficiency and reliability. Neuroimage. 2006;31(3):1116-1128. 16545965 10.1016/j.neuroimage.2006.01.015

[48] Zaid M Lansdown D Su F , et al. Abnormal tibial position is correlated to early degenerative changes one year following ACL reconstruction. J Orthop Res. 2015;33(7):1079-1086. 25721417 10.1002/jor.22867 PMC7238841

